# Difference and Analysis of Evaluating Psychological Monitors' Interview and Classmates' Being Interviewed About Suicide

**DOI:** 10.3389/fpsyg.2022.858903

**Published:** 2022-06-20

**Authors:** Qisheng Zhan, Tianyu Xia

**Affiliations:** ^1^School of Education, Tianjin University, Tianjin, China; ^2^Institute of Psychology, Tianjin University, Tianjin, China

**Keywords:** psychological monitors, interview about suicide, crisis intervention, suicide prevention, questionnaire survey

## Abstract

In recent years, suicide has become the leading cause of unnatural death among college students in China. Psychological monitors, as class cadres who manage affairs related to mental health within their classes, are critical in identifying and intervening in psychological crises among their classmates. In China, however, talking about death is a cultural taboo, and many mental health workers have expressed concern about their implementation of interviews about suicide with others. Generally speaking, interviews with suicidal classmates are usually conducted by professional psychological experts and psychological monitors (psychological monitors are non-professional peer counselors in China). Such classmates that have undergone both the aforementioned types of interviews were investigated in this paper. However, this paper focuses on two issues. Firstly, what are the perceptions of classmates who have been interviewed toward the experience of being interviewed by psychological monitors? Secondly, what are the psychological monitors' perceptions of their implementation of interviews about suicide with classmates? In this study, 1,664 classmates who had been interviewed and 1,320 psychological monitors were surveyed by means of an online questionnaire. The results showed that classmates who have been interviewed perceived their experience of being interviewed by a psychological monitor about suicide more positively than the psychological monitors who viewed their implementation of interviews about suicide with classmates. Among the classmates who have been interviewed, three categories of classmates have more positive perceptions of their experience of being interviewed by a psychological monitor about suicide. Category one is males. Category two is those who were willing to seek help from the psychological monitors. Category three is those who had a more correct attitude toward mental illness. Among the psychological monitors, three categories of psychological monitors have more positive perceptions of their implementation of interviews about suicide with classmates. Category one is those who have the experience of implementing interviews with their classmates. Category two is those who have received training. Category three is those who had a more correct attitude toward mental illness. Psychological monitors can interview classmates confidently, and the training of psychological monitors on mental health knowledge should be strengthened in universities.

## Introduction

Suicide is a recognized social and public health problem worldwide, with more than 800,000 deaths each year. According to the WHO, suicide is the second leading cause of death among people aged 15–29 years. In recent years, suicide was the first cause of death in the 15–34 age group in China, and the suicide rate among college students is about 0.2 per 1,000, which is the leading cause of unnatural death among college students (Chen et al., 2020). However, in the study of high-risk groups, the researchers unanimously found that young people with the highest risk of suicide were often the least likely to seek help from others (Miller, 2013). Therefore, it is crucial for schools, as the main place where college students live, to be proactive in suicide prevention and intervention. However, since ancient times, suicide has been the most humiliating form of human behavior. Suicide is a topic that is often considered taboo and often brings shame to the victim of suicide and his or her family. In culture, suicide is strictly prohibited, it is considered a crime in law, sin in religion, and insanity in medicine (Russell, 1968). In Chinese culture, death is a cultural taboo because it is believed that talking about it increases its likelihood of occurring. Therefore, Chinese people are also avoidant to talk about death (Wong et al., 2010). Concerns about suicide talk and intervention have also been confirmed in studies of numerous mental health workers. For example, Lakeman and Fitzgerald (2010) surveyed members of ethics committees who believed that by focusing subjects on suicidal thoughts and feelings, and by exposing them to distressing material, suicidal ideation may be exacerbated. Physicians also expressed their concerns about asking patients about suicidal thoughts. In a face-to-face interview with 170 family physicians, Stoppe et al. (1999) provided them with two written case profiles of depressed patients and asked them to diagnose the cases. The results showed that only 2.4% of the physicians were interested in their patients' suicidal thoughts, and when asked about it at the end of the interview, 19% of them said they would not talk about suicide. This shows that suicide awareness and prevention are not the most important issues for physicians, who are insecure about how to manage suicide. This was also shown in the studies of Bajaj et al. (2008) as well as Lang et al. (2009).

In 2004, the psychological monitors system was established in China. The psychological monitors system is related to peer counseling in Western countries, which can be traced back to the 1960s. As professional counseling staff in American schools were unable to meet the needs of students, some staff in American colleges and universities began to explore a new model of mutual support, namely students' psychological support organizations (Hu and Zhao, 2008). This model was earlier learned by China's Hong Kong and Taiwan and has achieved remarkable results (Li, 2009). In the 1980s, mental health education in Chinese colleges and universities was initially developed, and a large number of psychological counseling centers began to be established. However, when the enrollment of colleges and universities surged, the ratio of psychological teachers and college students in colleges and universities relatively became smaller, which cannot meet the requirements of students' mental health work, especially the early prevention and early detection of psychological crisis, so the system of psychological monitors was born (Xue et al., 2008). Since the establishment of the psychological monitors system, the number of psychological monitors in Chinese universities is estimated to have exceeded 748,000 (Zhan et al., 2018). In China, there are two psychological monitors (a male psychological monitor and a female psychological monitor) in each class (there are almost 30 college classmates in each small teaching class, and they are fixed during the period of 4 years). Psychological monitors are mainly responsible for holding mental health activities in the class and assisting the school mental health center to complete the mental health survey of classmates. Most importantly, they need to identify the psychological crisis of the classmates in the class and intervene in time. In the process of psychological crisis intervention, the early warning stage of psychological monitors plays an irreplaceable role. First of all, the psychological monitors get along with their classmates day and night, and psychological monitors can deeply observe and reflect the psychological states of their classmates in time. Their early warning work has bought valuable time for substantive crisis intervention in the later stage. At present, colleges and universities in China have established a psychological crisis intervention system of dormitory-class-college-school, and have started a psychological monitors supervision project (just as psychological counselors need to find supervisors when they encounter problems, psychological monitors can also turn to peer supervisors in time. This means that the psychological monitors are not solely responsible for early warning of crisis, but also have the support of professionals behind them. In the early warning of psychological crisis, the main function of the psychological monitors is the early identification of psychological crisis problems, while the later psychological crisis intervention is completed by full-time psychological counselors. In addition, there is a small age gap between the psychological monitors and their classmates, and they have the same concept of life and behavior. Therefore, psychological monitors can better accompany their classmates when their classmates are in trouble. In the process of accompanying the classmates, psychological monitors can also reduce or resolve the pressure and crisis of the classmates. One form of the psychological monitors' crisis intervention work is to proactively interview at-risk classmates within the class. However, researchers in China did not learn about the psychological monitors' attitudes toward their implementation of interviews about suicide with their classmates. Do they also hold negative perceptions about their implementation of interviews about suicide with their classmates? Based on the theory of planned behavior, the occurrence of individual behavior is composed of two stages. The first stage is the motivational stage, in which the overall behavioral intention of the individual is formed. The individual's behavioral intention is influenced by behavioral attitudes, subjective norms, and perceived behavioral control. The second stage is the implementation stage, in which the individual performs by developing a specific behavioral plan. During the motivation stage, behavior attitude is the positive or negative views held by individuals toward a certain behavior. Specifically, behavior attitude includes emotional attitude (whether people think the behavior is pleasant to carry out) and instrumental attitude (whether people think the behavior is advantageous). That is, if the individual thinks that the implementation of a certain behavior will bring positive results, then he will hold a positive attitude toward this behavior. If an individual thinks that the execution of an action brings negative results, then he has a negative attitude toward the behavior (Ajzen and Fishbein, 1980). It follows that individuals' attitudes toward their performance of a particular behavior influence their behavior. Therefore, in this study, we investigated the psychological monitors' attitudes toward their implementation of interviews about suicide with their classmates, which allowed us to speculate about their possible intervention behavior with at-risk classmates. Not only that, but we will also investigate the factors that influence the psychological monitors' attitudes toward their implementation of interviews about suicide with their classmates, so as to intervene in the psychological monitors' thoughts and thus facilitate their intervention behavior. Based on the above survey of mental health workers conducted by previous researchers, it is clear that some mental health workers feel that they are not competent to intervene with at-risk classmates and are therefore afraid to talk about suicide with others. It is hypothesized that some psychological monitors with lower levels of competence may be hindered in their implementation of interviews about suicide with classmates due to the avoidance of talking about suicide topics. Numerous scholars in China have studied the competencies of psychological monitors based on the iceberg model of competencies proposed by Spencer (Spencer et al., 1993). For example, Lai and Liu (2013) proposed a six-factor model of psychological monitors competency, Yi (2014) proposed a seven-factor model, and Zhan et al. (2021) proposed a five-factor model. However, regardless of the competency model proposed by the researcher, the importance of psychological knowledge and helping skills is valued. In this study, we explored whether the knowledge and experience of psychological monitors would have an impact on their attitudes toward their implementing interviews about suicide with classmates.

It is not enough just to explore the psychological monitors' attitudes toward their implementing interviews about suicide with classmates from their perspective, we also want to explore the perceptions of classmates toward the experience of being interviewed by psychological monitors, and thus we can test whether the concerns of the many mental health workers who ask others about suicide topics exist. Previous researchers have surveyed numerous respondents (patients, students, etc.) about their perceptions of talking about suicide with their doctors/counselors. Hahn and Marks (1996) had 293 patients in ongoing treatment at the Southeastern University Counseling Center fill out the Counseling Center Assessment Form and surveyed about their visits to suicide. According to the result, only 3% of respondents said that it was not a good idea for counseling centers' faculty to regularly assess respondents' past suicide attempts. This suggests that from the students' perspective, it is appropriate for the counseling centers' faculty to ask students about past suicide attempts on a regular basis and that students are not averse to the topic. In addition, Ballard et al. (2012) surveyed pediatric and adolescent patients in hospital emergency departments about their perceptions of the experience of being screened for suicide by their doctors. Ninety-six percent of people support being asked by a doctor about suicide, and they believe they can gain understanding and attention by being asked by a doctor. Not only that, but young patients in the emergency room (Elizabeth et al., 2013) and adult inpatients in general hospitals (Snyder et al., 2016) support the need for screening inpatients for suicide risk. Finally, previous researchers have also examined the effects of subjects' participation in suicide-related questionnaire screenings (Gould et al., 2005; Deeley and Love, 2010; Robinson et al., 2011; Mathias et al., 2012; Whitlock et al., 2013; Law et al., 2015; Beurs et al., 2016; Harris, 2016; Crawford et al., 2018; Hom et al., 2018), interviews (Bjärehed et al., 2013; Rahel et al., 2014; Theodore et al., 2019), and studies including suicide-related content (Reynolds et al., 2006; Taylor et al., 2010; Cukrowicz et al., 2012; Rivlin et al., 2012; Gibson et al., 2014; Hasking et al., 2015; Cha et al., 2016; Rebecca et al., 2016; Lockwood et al., 2018) on their emotions and suicidal ideation through empirical studies. It was found that most people agreed with the idea of being asked about suicide by others and also thought it would be beneficial to talk about suicide with others. In China, however, no researchers have investigated classmates' perceptions toward the experience of being interviewed by others. We believe that there is a need to explore the perceptions of classmates toward the experience of being interviewed by psychological monitors in the Chinese cultural context. In this study, we only investigate the perceptions of classmates who have been interviewed about suicide by psychological monitors and professional psychological experts, because we believe classmates have the experience of having been interviewed and therefore have a more realistic view of being interviewed by psychological monitors and professional psychological experts. In addition, to enhance their emotional experience in suicide interviews, we also explore what characteristics of theirs would have an impact on their interview experience. Although previous researchers have not studied the effectiveness of counseling by psychological monitors, some researchers have studied the effectiveness of counseling for psychological counselors. Individuals fall under the category of psychological help whether they receive help from counselors or psychological monitors. Therefore, this study investigates the factors that influence the effectiveness of individuals receiving interviews in terms of factors that affect the effectiveness of counseling. Previous researchers have conducted systematic research on the effects of participation in counseling. Researchers have studied this area through 3 stages: process research, effect research, and process-effect research (Jiang and Shu-Jing, 2010). The research on counseling process-effectiveness has divided the factors affecting counseling effectiveness into two categories: in-session factors and between-session factors. The factors that influence the effectiveness of counseling have been divided into two main categories: in-talk and between-talk factors. The elements in the session that affect the effectiveness of counseling are the counselor's verbal response, client's own behavior, working alliance, etc. Inter-session factors that influence the effectiveness of counseling are inter-session experience, sudden benefits, counseling expectations, etc. (Wang and Zhou, 2017). The individual's consultation expectations include the idea that the individual is willing to conduct the consultation, the individual's openness in the consultation, etc. (Tinsley et al., 1980). In this study, we focused on helper behavior in the intra-meeting factors and counseling expectations in the inter-meeting factors.

In summary, this study explores the dual perspectives of psychological monitors and classmates. The findings derived from this study can provide empirical evidence for interventions by psychological members and at-risk college students. This study has important implications for the management of mental health efforts in colleges and universities.

## Materials and Methods

### Participants

The survey was conducted on the Internet, and the questionnaire was posted online through the National Psychological Monitors' Work Platform, and then the questionnaires were forwarded to each faculty and class by the teacher in charge of managing the psychology monitors at each school. In this survey, the contents of the questionnaire filled out by the psychological monitors as well as the classmates were different. The duration of this online survey was 3 days. Informed consent was obtained from the participants before filling out the questionnaire, and participants who were not willing to fill out this questionnaire would directly exit the questionnaire filling interface. The final participants of this study were psychological monitors, classmates (non-psychological monitors), and full-time psychological teachers from major universities in China (in this study, the information of questionnaires completed by teachers was not counted). A total of 16,631 participants participated in the survey, and 1,046 refused to fill out the questionnaire. We do not know their specific information because the participants refused to fill out the questionnaire. The final number of participants in the survey was 15,585, including 57 full-time psychology teachers, 1,320 psychological monitors, and 14,208 classmates (non-psychological monitors). Among the psychological monitors, there were 175 (13.3%) junior college students, 1,110 (84.1%) undergraduate students, 33 (2.5%) master students, and 2 (0.2%) doctoral students; 462 (35%) male students and 858 (65%) female students; 422 (32%) only- children and 898 (68%) non-only-children; 718 rural students (54.4%), and 602 urban students (45.6%); the average age was 20.08 years. In this study, we used a questionnaire to survey a total of 14,208 students. Of these 14,208 classmates, 1,664 (11.7%) were interviewed about suicide, and 12,544 (88.3%) were not interviewed. We only analyzed the perceptions of classmates who had been interviewed. Among classmates who have been interviewed, 726 (43.6%) were junior college students, 920 (55.3%) were undergraduate students, 10 (0.6%) were master's students, and 8 (0.5%) were doctoral students; 667 (40.1%) were male students and 997 (59.9%) were female students; 460 (27.6%) were only- children and 1,204 (72.4%) were not only-children; 1,037 (62.3%) were rural students and 627 (37.7%) were urban students. The average age of the participants was 19.71 years.

### Measures

#### Questionnaire for Psychological Monitors

In order to study the psychological monitors' perceptions of their implementation of interviews about suicide with classmates, we developed our own questionnaire for psychological monitors. The psychological monitors were surveyed mainly from the following aspects: (1) Demographic information. This included the gender of the psychological monitors, psychological monitors' degree, whether they were only-children, and the location of their families. (2) The knowledge reserve and working experience of the psychological monitors. ① Whether they have conducted suicide-related interviews with their classmates. ② Training they received after serving as a monitor. There are four options for this question: a. Systematic training(Psychological monitors participated in a semester of systematic training, including a variety of psychological theoretical knowledge and practical skills);b. More than one training(Psychological monitors participated in special training, including academic problems, love problems, family problems, dormitory relationship problem**s**, employment problem, and crisis problems);c. Plan to participate in training(The school has a training program, but the psychological monitors ha**ve** not yet participated in the training);d. Untrained(The psychological monitors did not participate in the training). (3) Psychological monitors' attitudes toward the implementation of interviews about suicide with classmates. As shown earlier, if an individual thinks that the implementation of an action will bring positive results, then he will have a positive attitude toward this behavior. If an individual thinks that the implementation of an action will bring negative results, then he will have a negative attitude toward this behavior. Therefore, we used a question which is to ask the psychological monitors what the expected results of the implementation of interviews about suicide with classmates are. The title is: In my opinion, when I interview a classmate about suicide, I think his mood will: ① get better; ② stay the same; ③ get worse. We also had them fill in specific reasons in text form.

#### Questionnaire for Ordinary Classmates

In order to study the perceptions of classmates who had been interviewed toward the experience of being interviewed by psychological monitors, we developed our own questionnaire. The classmates were surveyed mainly in the following aspects: (1) Demographic information. This included their gender, their degree, whether they were only-children, and family location. (2) Experiences of classmates seeking psychological help. ① Whether they have been interviewed about suicide. ② Whether they are willing to seek help from the psychological monitors when they have psychological problems or confusion. (3) Classmates' perceptions toward the experience of being interviewed by psychological monitors. The topic is: In my opinion, when I was interviewed by the psychological monitor about suicide, my mood would ① get better; ② stay the same; ③ get worse. We also had them fill in specific reasons in text form.

#### Questionnaire on Public Attitudes Toward Mental Illness

Finally, we had both the classmates and the psychological monitors fill out the Public Attitudes Toward Mental Illness Questionnaire recommended by the former Ministry of Health, which has a total of 12 questions. The main contents include public perceptions and beliefs about mental illness and people with mental illness and public motivations, thoughts, and reactions in establishing and maintaining relationships with people with mental illness. This questionnaire is scored on a scale of 1–5, from fully agree to fully disagree. Items 5, 6, 7, 9, 11, and 12 are reverse scored, and the final total score is calculated. The total score is the mental illness-related attitudes questionnaire score. The higher they scored, the more positive the attitude toward mental illness (Xi et al., 2014).

### Data Analysis

First, we conducted a frequency analysis of the psychological monitors' perceptions of their implementation of interviews about suicide with classmates. In addition, we conducted a frequency analysis on the perceptions of classmates who have been interviewed toward the experience of being interviewed by psychological monitors.

We then used a chi-square test and a one-way ANOVA to explore the factors that influenced the psychological monitors' perceptions of their implementation of interviews about suicide with classmates.

In addition, we also used a chi-square test and a one-way ANOVA to analyze the factors that influenced the perceptions of classmates who have been interviewed toward the experience of being interviewed by psychological monitors.

Finally, we coded and organized the textual information about the perceptions of the classmates as well as the psychological monitors, extracting the relevant interview-related concepts expressed in each sentence. In order to correctly reflect the original meaning of the respondents, the original words were used to distill the initial concepts as much as possible. The frequency of the collated positive or negative vocabulary was counted, and the counted vocabulary categories were grouped together.

### Ethical Considerations

The Psychological Monitors Ethics Committee approved the study. All the respondents filled out informed consent before filling out the questionnaire.

The procedures of this study complied with the provisions of the Declaration of Helsinki regarding research on human participants.

## Results

### Comparison of Perceptions of Interviews About Suicide

Table 1 shows that 21.2% of the psychological monitors thought that their implementation of interviews about suicide with classmates would make the classmates ' mood better(referred to as “psychological monitors' views” in the table). Results show that 44.8% of the psychological monitors thought that their implementation of interviews about suicide with classmates would not make the respondents' mood change, and 34.0% of the psychological monitors thought that their implementation of interviews about suicide with classmates would make the respondents' mood worse.

**Table 1:** Psychological monitors' views on interviews about suicide.

**Psychological monitors' views**	**Frequency**	**Percentage**
Getting better	280	21.2
No change	591	44.8
Getting worse	449	34.0
Total	1,320	100.0

Table 2 shows that 25.2% of the classmates thought that their mood (referred to as “classmates' self-evaluation” in the table) would change for the better when they were interviewed by a psychological monitor, 67.1% thought that being interviewed by a psychological monitor would not change their mood, and the percentage of those who thought that being interviewed by the psychological monitor would make their mood worse was 7.7%.

**Table 2:** The views of the classmates who have participated in the interviews about suicide.

**Classmates' self-evaluation**	**Frequency**	**Percentage**
Getting better	419	25.2
No change	1,117	67.1
Getting worse	128	7.7
Total	1,664	100.0

Taken together, this data suggests that psychological monitors held an overly negative view toward their implementation of interviews about suicide with classmates.

### Factors Influencing Perceptions of Interviews About Suicide

#### Demographic Factors

First, we analyzed the psychological monitors. The analysis of variance in Table 3 shows that there were no significant differences in the psychological monitors' perceptions of their implementation of interviews about suicide with classmates, regardless of their gender, family location, whether they were an only-child, their degree.

**Table 3:** Analyses of psychological monitors' differences of viewpoints on the class students interviewed with suicide from the perspective of multiple identities.

	**Getting better**	**No change**	**Getting worse**	**χ^2^**	***p***
Gender				2.417	0.299
Male	109(23.6%)	200(43.3%)	153(33.1%)		
Female	171(19.9%)	391(45.6%)	296(34.5%)		
Family location				2.883	0.237
Rural areas	141(19.6%)	322(44.9%)	255(35.5%)		
City	139(23.1%)	269(44.7%)	194(32.2%)		
Only-child				2.320	0.314
Yes	79(18.7%)	194(46.0%)	149(35.3%)		
No	201(22.4%)	397(44.2%)	300(33.4%)		
Students'					
Degree				11.183	0.052
College students	24(13.7%)	86(49.1%)	65(37.2%)		
Undergraduate	244(22.0%)	492(44.3%)	374(33.7%)		
Master	11(33.3%)	12(36.4%)	10(30.3%)		
Doctor	1(50.0%)	1(50.0%)	0(0.0%)		
Interviewed					
Other students				29.470	0.000
Yes	107(25.8%)	209(50.5%)	98(23.7%)		
No	173(19.1%)	382(42.2%)	351(38.7%)		
Training				13.051	0.042
Systematic Training	131(26.0%)	207(41.1%)	166(32.9%)		
More than one training	123(19.0%)	303(47.0%)	219(34.0%)		
Plan to participate in training	13(14.9%)	42(48.3%)	32(36.8%)		
Untrained	13(15.5%)	39(46.4%)	32(38.1%)		

We then analyzed the classmates. The analysis of variance in Table 4 shows that there were no significant differences in the classmates' perceptions of the experience of being interviewed for suicide by the psychological monitors, regardless of the classmates' home location, whether they were an only-child, or the classmates' degree.

**Table 4:** Analysis of differences in interviews about suicide among different categories of classmates.

	**Getting better**	**No change**	**Getting worse**	**χ^2^**	***p***
Gender				38.275	0.000
Male	220(33.0%)	409(61.3%)	38(5.7%)		
Female	199(20.0%)	708(71.0%)	90(9.0%)		
Family location				1.240	0.538
Rural areas	261(25.2%)	702(67.7%)	74(7.1%)		
City	158(25.2%)	415(66.2%)	54(8.6%)		
Only-child				0.222	0.895
Yes	118(25.7%)	305(66.3%)	37(8.0%)		
No	301(25.0%)	812(67.4%)	91(7.6%)		
Students' degree				5.731	0.390
Junior college students	189(26.0%)	491(67.6%)	46(6.4%)		
Undergraduate	225(24.5%)	615(66.8%)	80(8.7%)		
Master	2(20.0%)	7(70.0%)	1(10.0%)		
doctor	3(37.5%)	4(50.0%)	1(12.5%)		
Willingness to asking for help				89.801	0.000
Yes	373(29.8%)	813(65.0%)	64(5.2 %)		
No	46(11.1%)	304(73.4%)	64(15.5%)		

However, there were significant differences in the perceptions of classmates by gender. The further judgment shows that a higher percentage of males than females believe that being interviewed by a psychological monitor for suicide will make them feel better. A lower percentage of males than females believed that being interviewed by a psychological monitor for suicide would make them emotionally worse.

#### The Attitude Toward Mental Illness

Among psychological monitors, a one-way ANOVA revealed a significant difference in attitudes toward mental illness among psychological monitors with different views on their implementation of interviews about suicide with classmates, *F* = 8.639, *p* < 0.01. Therefore, using a Bonferroni correction for all possible two-by-two comparisons of these variables, those who believed that their implementation of interviews about suicide with classmates' mood worse had a scored significantly lower than those who believed that their implementation of interviews about suicide made the classmates' mood better as well as unchanged (see Table 5). It indicates that the more correct the psychological monitor's attitude toward mental illness, the better the perception of their implementation of interviews about suicide with classmates.

**Table 5:** The relationship between the attitude of classmates/psychological monitors toward mental illness and their emotional changes in self-assessment.

**Self-evaluation**	**Getting better**		**No change**		**Getting worse**		
	***M***	***SE***	***M***	***SE***	***M***	***SE***	***F***	***p***
Attitude toward								
mental illness (classmates)	37.652	0.270	38.375	0.166	35.727	0.489	14.198	0.00^a^^b^
Attitude toward								
mental illness (Psychological monitors)	38.757	0.373	37.843	0.257	36.817	0.295	8.639	0.00^a^^b^

*M = estimated marginal mean (estimated marginal mean);*

a *indicates that there is a significant difference between the group with worse mood and the group with better mood;*

b *indicates that there is a significant difference between the group with worse mood and the group with unchanged emotion*.

Among classmates, a one-way ANOVA showed that there were significant differences in attitudes toward mental illness among those who held different views about being interviewed for suicide by psychological monitors, *F* = 14.198, *p* < 0.01. Therefore, using a Bonferroni correction for all possible two-by-two comparisons of these variables, classmates who believed that being interviewed by a psychological monitor for suicide made their mood worse scored significantly lower on attitudes toward mental illness than classmates who believed that being interviewed by a psychological monitor for suicide made their mood better as well as unchanged. It indicates that the more correct their attitude toward mental illness is, the better their perception of the experience of being interviewed by psychological monitors (see Table 5).

#### Other Factors

Among psychological monitors, a higher percentage of psychological monitors who had interviewed other classmates believed that their implementation of interviews about suicide with classmates would make the interviewee feel better than those who had not interviewed other classmates. A lower percentage of psychological monitors who had interviewed other classmates believed that their implementation of interviews about suicide with classmates would make the interviewee worse than those who had not interviewed other classmates. However, overall, the percentage of psychological monitors who thought that their implementation of interviews about suicide with classmates would make them feel worse was higher than 20%, regardless of whether they had interviewed or not. In addition, there was a significant difference between the different training received by the psychological monitors and their perceptions of their implementation of interviews about suicide with classmates. Table 3 shows that the more systematic training the psychological monitors received, the higher the percentage who believed that their implementation of interviews about suicide with classmates would make them feel better.

Among the classmates, those who were willing to seek help from the psychological monitors when they were confused had more positive perceptions toward the experience of being interviewed for suicide by the psychological monitors than those who were not willing to seek help. Further judgment shows that a higher percentage of classmates who were willing to seek help believed that being interviewed by a psychological monitor for suicide would make them feel better than those who were not willing to seek help. The percentage of classmates who were willing to seek help who thought that being interviewed by a psychological monitor for suicide would make them feel worse was lower than those who were not willing to seek help (shown in Table 4).

### Textual Analysis of Perceptions of Interviews About Suicide

#### The Psychological Monitor's View

A total of 194 of the 280 people who had positive views about their implementation of interviews about suicide with classmates expressed their opinions. The vocabulary of psychological monitors' comments on their implementation of interviews about suicide with classmates is shown in Table 6.

**Table 6:** Psychological monitors' positive view of suicide interviews.

**Entry of the word**	**Frequency**	**Entry of the word**	**Frequency**
Saying everything	58	Listening	10
Enlightenment	26	Venting emotions	8
Finding a solution	19	Relieving stress	8
Bringing positive cues	18	Empathy	8
Regulating emotions	16	Feeling noticed	6
Understanding the problem	13	Getting Help	6
Facing up to the problem	11	Feeling cared for	5
Releasing stress	10	Helping classmates to share	3
Relieving negative emotions	10	Preventing problems	2
		Companionship	1

Their following views were mainly summarized. ① The **classmates** were able to confide in the interviews, regulate, vent and relieve their negative emotions, and were able to release and relieve their stress. ② The **classmates** were able to face their problems head-on during the interviews, understand their problems, and be able to prevent their serious problems from occurring. ③ The **classmates** were able to feel cared for, noticed, heard, and accompanied during the interviews. ④ During the interview, the psychological monitors were able to guide them, help them share their problems, help the interviewees find ways to solve their problems, and bring them a positive influence.

Of the 449 individuals who had a negative view of their implementation of interviews about suicide with classmates, 325 expressed their opinion. The vocabulary of psychological monitors' comments on their implementation of interviews about suicide with classmates is shown in Table 7. Their following views were mainly summarized. ① Psychological monitors consider suicide to be a private, heavy, negative, sensitive, serious, and bad topic. They think that classmates do not want to talk about such topics in depth. ② They believe that such topics can stimulate the emotions of their classmates, evoke their own inner turmoil, or similar experiences. ③ They believe that interviewing **classmates** about suicide will have a tendency to induce suicide and will make classmates who want to commit suicide want to do so even more. ④ They are not good at these types of topics and are afraid to interview them.

**Table 7:** Psychological monitors ' negative view of suicide interviews.

**Entry of the word**	**Frequency**	**Entry of the word**	**Frequency**
Triggering negative emotions	106	Negative topics	16
Heavy Topics	37	Fear of talking about	14
Interviewer's reluctance to discuss in-depth	32	Inducing interviewees to commit suicide	8
Stimulating the interviewee	28	Interviewees with	6
Sensitive topics	24	psychological problems	
Evoking negative thoughts	23	Serious Topics	6
Bad topics	18	Privacy	5
		Not good at	4

#### Classmates' Views

A total of 186 of the 419 people who had positive views about being interviewed for suicide by a psychological monitor expressed their opinions. The vocabulary of their comments on being interviewed for suicide by psychological monitors is shown in Table 8 below.

**Table 8:** Positive perceptions of suicide interviews by classmates.

**Entry of the word**	**Frequency**	**Entry of the word**	**Frequency**
Saying everything	90	Getting Help	8
Getting communication	20	More positive	5
Liking the psychological monitors	19	Changing of mind	5
Relieving negative emotions	18	Being understood	3
Getting cared for	18		

We mainly summarized their views as follows. ① In the interview they can confide, talk, and communicate their ideas. ② During the interviews, they felt cared for and felt that someone was willing to help them. ③ During the interview, one's original thoughts will change and become more positive and optimistic. ④ They think that psychologists are good people to talk to. They think that psychological monitors are good people to talk to because they are similar to them in age, they can communicate with each other openly, and they are very relatable people.

Of the 128 people who had a negative view of being interviewed for suicide by a psychological monitor, 70 expressed their opinion. The vocabulary of their comments is shown in Table 9. Their following main views were summarized. ① They do not trust, are not familiar with, do not like, or even hate the psychological monitors. ② They feel that they do not understand them and feel that the interview is useless. ③ They do not want to let more people know, do not want to tell their classmates what they think, and cannot trust their classmates to protect their privacy. ④ They believe that talking about this topic will induce negative emotions in themselves.

**Table 9:** Negative perceptions of classmates about suicide interviews.

**Entry of the word**	**Frequency**	**Entry of the word**	**Frequency**
Triggering negative emotions	17	Not familiar with the psychological monitors	3
Topics I don't want to talk about	9	Not being understood	3
Privacy	7	Serious Topics	3
Things you don't want others	6	Sensitive Topics	3
to know		Heavy Topics	2
Distrust	6	Not useful	2
Don't want to communicate	5		
Dislike for the psychological monitors	4		

## Discussion

### Psychological Monitors' and Classmates' Views on Interviews About Suicide

Our study showed that only 7.7% of the classmates thought that being interviewed by a psychological monitor for suicide would make their mood worse, which means that most of the classmates thought that being interviewed by a psychological monitor for suicide would not have a negative effect on their mood. Although we did not use a rigorous experimental procedure for having psychological monitors interview classmates, our investigation also corresponds to the results of previous empirical studies of interviews about suicide conducted with a variety of subjects. We were surprised that the psychological monitors' perceptions were very different from the perceptions of the classmates. The percentage of psychological monitors who believed that their implementation of interviews about suicide with classmates would make the interviewees' moods worse exceeded the percentage of classmates who believed that their interview by a psychological monitor would make their moods worse by 25%, indicating that psychological monitors have an overly negative view of their implementation of interviews about suicide with classmates. By compiling the texts of the psychological monitors, we found that their concerns were mainly that they were not good at interviewing and that their implementation of interviews about suicide with classmates would cause intense emotional changes in the interviewees or induce suicidal tendencies in the interviewees. However, these concerns are overstated because many people who have been interviewed by others in the past have liked the interview because it was therapeutic, with the interview process being cathartic, and have said that the interview provided them with the opportunity to talk deeply about their experiences, facilitated their self-expression, and that they were listened to by others (Jorm et al., 2007; Clark et al., 2012; Rivlin et al., 2012; Biddle et al., 2013). Based on the theory of planned behavior, psychological monitors' perceptions of their implementation of interviews about suicide with classmates influence their behavior in their implementation of interviews about suicide with classmates. Thus, psychological monitors who believe that their implementation of interviews about suicide with classmates will make the interviewee's mood worse may not initiate interviews about suicide with their classmates. Therefore, we want to examine the influencing factors that affect the psychological monitors' perceptions of their implementation of interviews about suicide with their classmates. By starting with the influencing factors, it is possible to intervene in classmates' perceptions.

### Factors Influencing Psychological Monitors' Perceptions of Interviews About Suicide

In this study, we explore whether these factors have an impact on the psychological monitors' attitudes toward their implementation of interviews about suicide with classmates from the perspective of their mental health knowledge and hands-on experience. From the perspective of psychological monitors' practical experience, our findings revealed that a higher percentage of psychological monitors who had interviewed other classmates believed that their implementation of interviews about suicide with classmates would make the interviewee feel better than those who had not interviewed other classmates. It is evident that the more hands-on experience psychological monitors have, the better their perceptions of their implementation of interviews about suicide with classmates. The importance of hands-on experience for their competency has been confirmed in previous studies (Hu, 2018). However, whether they had conducted interviews with other classmates, the psychological monitors' negative perceptions of their implementation of interviews about suicide with classmates exceeded 20 percentage points, indicating that even those psychological monitors who had interviewed classmates were still not very sure about the effectiveness of the interviews. Therefore, in the future training for psychological monitors, we should arrange more courses on crisis intervention to strengthen the confidence of psychological monitors to conduct crisis intervention. We then looked at the impact on the psychological monitors' attitude toward their implementation of interviews about suicide with classmates in terms of the mental health knowledge they possessed. Our findings found that the psychological monitors' attitudes toward mental illness were associated with their attitudes toward their implementation of interviews about suicide with classmates. The reason for this is that psychological monitors' attitudes toward mental illness are part of their mastery of mental health learning, and the higher an individual's mastery of mental health learning, the higher their level of competency in their job. As a result, they then have a good attitude toward their implementation of interviews about suicide with classmates. Therefore, it is crucial for psychological monitors to have knowledge of mental health. Finally, the study was discussed in terms of the training received by the psychological monitors. The importance of training for psychological monitors to acquire mental health knowledge and hands-on experience has been identified in previous studies (Yu et al., 2020).

The results of our study found that the more systematically trained the psychological monitors were, the higher the percentage of them believed that their implementation of interviews about suicide with classmates would make the respondent feel better.

Thus, it is clear that the study of the above influencing factors is important to guide the work of future psychological monitors. We need to provide systematic training for psychological monitors, including not only training on knowledge related to mental illness but also training on practical skills. In addition, psychological monitors should be trained in other aspects. In this way, the psychological monitors can constantly gain and improve from theory and practice. In the training of theoretical knowledge, psychological monitors are trained mainly in abnormal psychology, developmental psychology, social psychology, psychological counseling, and psychotherapy. In the practical skills training, the psychological monitors are trained mainly in the aspects of crisis identification technology, listening technology, support technology, and referral technology. In the form of training, psychological monitors should be trained not only by traditional teaching methods, but also by situational simulation and role-playing. In addition, with the introduction of the internet, the way of internet training has also been introduced into the curriculum of psychological monitors. Therefore, it is necessary to fully cover the online and offline training of psychological monitors. If the psychological monitors still have no confidence in mental health work after participating in the training, they can ask the psychological monitors' supervisor or the head teacher in this class (the teacher in charge of crisis management) for help at any time. This can not only reduce their burden on mental health work, but also improve their mental health work skills. Moreover, according to the decision theory of helping behavior proposed by Burnstein et al. (1994), helping people is a responsible social cognitive, and rational decision-making process. The first stage is noticing the event and interpreting it correctly (perceiving the need to help), the second stage is determining personal responsibility, the third stage is weighing the pros and cons of the behavior, and the fourth stage is determining personal competence and behavioral style. Psychological monitors, as class leaders who manage mental health matters within the class, who notice a crisis event and believe that they also have a responsibility to help and can afford to do so, do not end up doing so because they do not know how to help or are unable to do so. This theory further illustrates the importance of training in mental health knowledge and practical skills. It is only when psychological monitors acquire more knowledge and skills in psychology that their level of competency increases and they can hold a good attitude toward their implementation of interviews about suicide with classmates, thus promoting their helping behaviors.

### Factors Influencing the Classmates' Perceptions of Interviews About Suicide

In previous studies of counseling, the factors that influence the effectiveness of counseling have been divided into two categories: factors during the sessions and factors between sessions. In this study, we focused on helper behavior and counseling expectations among the factors in the talks. Counseling expectations include many factors, such as the helper's motivation for counseling, the helper's openness in counseling, etc. (Tinsley et al., 1980). From the perspective of the help-seekers themselves, among the classmates who had been interviewed about suicide, a higher percentage of men than women believed that interviews about suicide would make them feel better. That is, men had a more positive view of interviews about suicide than women. Rivlin's previous findings from a study of interviews about suicide with prisoners had similar findings, which may be due to the different attitudes of boys and girls toward the nature of suicidal behavior (Wang et al., 2005). As a result, psychological monitors should pay special attention to the emotional care of female classmates in their future crisis intervention work. From the perspective of the motivation of those who sought help, a higher percentage of classmates who were willing to seek help from a psychological monitor when they were confused believed that an interview about suicide with a psychological monitor would make the interviewee feel better than those who were not willing to seek help. The willingness of the classmates to seek help from the psychological monitors when they are confused indicates that they have a good motivation to seek help. People who have good motivation to seek help have higher expectations of counseling and therefore are likely to have good counseling outcomes. It follows that we can intervene with our classmates from the perspective of their motivation to seek help. Finally, the classmates' openness in the interviews also influenced their perceptions of interviews about suicide. The results of our study found that classmates who believed that participating in interviews about suicide made their mood worse scored significantly lower on attitudes toward mental illness than classmates who believed that interviews about suicide made their mood better as well as unchanged. College students' attitudes toward mental illness reflect, to some extent, their sense of morbidity about receiving psychological help (Xi et al., 2014), and this morbidity can affect classmates ' openness in counseling. If classmates are not open to sharing their questions and ideas during the interview, then it will affect their experience during the interview. In summary, we have studied the emotional experiences of the classmates during the interviews from the perspective of the helpers themselves as well as counseling expectations. The study of the above influencing factors has important implications for classmates and psychological monitors to conduct interventions. From the psychological monitors' perspective, their classmates ' expectations are part of their counseling expectations. In the selection process, if the selected psychological monitors have good interpersonal skills and can get along with the classmates, then the classmates will be willing to seek psychological help from them, and they will also be able to achieve better results (Tian, 2015). From the perspective of classmates, mental health knowledge can be conveyed to them in the form of mental health courses for college students to reduce their sense of morbidity about seeking psychological help and to raise their expectation of seeking help from psychological monitors.

### Textual Analysis of Perceptions of Suicide Interviews

By compiling the views of the psychological monitors and classmates, we were also able to draw some insights for future work. Among those psychological monitors who had a negative view of their implementation of interviews about suicide with classmates, the main comments they made are shown below. First, they were avoidant about talking about suicide. Second, they have an overly negative view of their implementation of interviews about suicide with classmates. Finally, many felt that they were not competent to do the job of interviewing about suicide. From the above, it is clear that in future training efforts, we need to pass on to them not only the knowledge of crisis intervention but also the empirical studies related to interviews about suicide that have been conducted by scholars in the past, as a way to strengthen their confidence in crisis management.

Among those classmates who had negative views about being interviewed for suicide by the psychological monitors, they mainly expressed the following opinions. First, due to trust and familiarity issues, they do not want to communicate with the psychological monitors about the topic and believe that the interview is not useful for them. Second, some classmates like to hide when they encounter psychological problems and do not want to deal with them actively. From the above, the selection of psychological monitors should be done by selecting those who have a high degree of trust in the class. In addition, psychological monitors should conduct more mental health activities in the class and take the initiative to communicate and intervene with those who have psychological problems. This will enhance the sense of trust and familiarity with the classmates. Finally, based on ironic process theory, suppression of unwanted thoughts, especially suicidal thoughts, would be associated with higher frequency and greater intensity of suicidal ideation (i.e., the rebound effect) (Pettit et al., 2010). Therefore, in the future mental health education work for classmates, it is important to teach them the correct way to deal with psychological problems. Do not let the classmates bury their psychological problems in their hearts and prevent them from developing into serious crises.

## Advantages and Limitations

The role of the psychological monitors in the study was introduced in this study. We explored the psychological monitors' perceptions' of their implementation of interviews about suicide with classmates, which serves as a groundwork for similar future studies. In addition, the factors influencing the classmates' perceptions of being interviewed by the psychological monitors and the psychological monitors' perceptions of their implementation of interviews about suicide with classmates were examined this study. On the one hand, future researchers can use this study as a basis for an empirical study of psychological monitors conducting interviews about suicide with classmates. Future researchers should examine the factors that influence the effectiveness of psychological monitors' interviews from the perspective of more characteristics of psychological monitors. On the other hand, future psychology research workers can better promote suicide intervention by intervening with psychological monitors and classmates from these factors.

However, this study also has limitations in that we only administered a questionnaire to the subjects to ask them about their perceptions of being interviewed for suicide by a psychological monitor, and did not conduct the study by doing an experiment. Future researchers could further explore this topic with an experimental study of both psychological monitors and classmates.

## Conclusion

This study revealed that psychological monitors had more negative perceptions of their implementation of interviews about suicide with classmates. According to our research results, this may be caused by the psychological monitors' lack of relevant psychological knowledge and psychological assistance skills. Therefore, professional psychological trainers in colleges and universities should carry out professional and systematic training for psychological monitors. Professional psychological trainers should not only require psychological monitors to master the basic knowledge of college students' mental health, learn to identify the signals of psychological crisis, but also require them to learn some methods and skills of helping others and communicating.

Specifically, first, professional trainers should teach psychological monitors theoretical knowledge, such as psychological counseling and treatment, knowledge of common psychological problems of college students (learning problems, interpersonal problems, etc.). Secondly, the professional trainers should teach the psychological monitors the basic skills of helping others, such as listening technology, support technology, and so on. Finally, we should not only adopt the traditional teaching method, but also use experiential training, thematic training, activity training, and other ways to train psychological monitors. With the development of the Internet, online training should also be popularized in colleges and universities.

The psychological monitors get along with their classmates day and night, identify their psychological state at the first time, and play an irreplaceable role in the four-level protection network of psychological crisis in colleges and universities. Behind the psychological monitors, there are supervisors, college-level psychological professionals, and school-level psychological professionals. This means that the psychological monitors are not solely responsible for early warning signs of crisis, but also have the support of professionals behind them. Therefore, we should dispel their worries about psychological crisis identification and enable the psychological monitors to continue to play a role in suicide prevention.

## Data Availability Statement

The raw data supporting the conclusions of this article will be made available by the authors, without undue reservation.

## Author Contributions

QZ did the preliminary data collection work. TX and QZ jointly completed the writing and revision of the paper. Both authors contributed to the article and approved the submitted version.

## Funding

This study was supported in part by the grants from Tianjin Education Committee Scientific Research Program Special Task Key Project Research on competency model of psychological monitors implementing peer psychological service (Grant No. 2020ZXXL-GX19G) and Tianjin Education Committee Social Science Key Project Standardization construction and practice of mental health in colleges and universities (Grant No. 2020JWZD13).

## Conflict of Interest

The authors declare that the research was conducted in the absence of any commercial or financial relationships that could be construed as a potential conflict of interest. The reviewer ZL declared a past collaboration with the author QZ to the handling editor.

## Publisher's Note

All claims expressed in this article are solely those of the authors and do not necessarily represent those of their affiliated organizations, or those of the publisher, the editors and the reviewers. Any product that may be evaluated in this article, or claim that may be made by its manufacturer, is not guaranteed or endorsed by the publisher.
